# Protocol for the “motor-brain development” trial: effects of motor-focused exercise training on brain function and gross motor skills in preschool children

**DOI:** 10.3389/fped.2026.1777657

**Published:** 2026-04-13

**Authors:** Xin-Chen Wang, Bo Zhou, Yong-Hong Bao, Hua Xia

**Affiliations:** College of Physical Education, Hunan Normal University, Changsha, China

**Keywords:** electroencephalography, executive function, gross motor skills, neurocognitive development, physical education, preschool children

## Abstract

**Purpose:**

Early childhood is a critical period for both motor and neurodevelopment, yet less is known about whether behavioral improvements following motor-focused physical education are accompanied by concurrent functional neural indicators. This protocol describes a cluster-randomized trial evaluating the effects of a semester-long motor-focused exercise program on gross motor competence and exploratory neurodevelopmental outcomes in preschool children.

**Methods:**

This two-arm, cluster-randomized controlled trial will recruit approximately 110 children aged 4–6 years from kindergartens in Changsha, China. Classes will be randomized 1:1 to either an intervention group receiving structured motor-focused physical education or a control group receiving ordinary physical education. The intervention will be delivered three times weekly for 30–40 min over 16 weeks, emphasizing locomotor and object-control skills through progressive, game-based activities. The primary outcome will be gross motor competence measured by the Test of Gross Motor Development–Third Edition (TGMD-3). Secondary outcomes will include portable electroencephalography (EEG) measures of resting-state and task-related brain activity associated with inhibitory control and attentional regulation. Optional measures will include parent- and teacher-reported questionnaires of executive function and behavioral adjustment. Group-by-time effects will be analyzed using linear mixed-effects models under an intention-to-treat framework.

**Discussion:**

This trial will examine whether structured motor-focused physical education is associated with improvements in gross motor competence and concurrent functional neural indicators in preschool settings. Findings will contribute incremental evidence to the developmental literature linking motor and neurocognitive processes and may inform future research on scalable approaches to support early childhood development.

**Clinical Trial Registration:**

https://clinicaltrials.gov/study/NCT07307742 identifier NCT07307742.

## 1 Introduction

Early childhood represents a critical period for pediatric neurodevelopment, during which foundational motor, cognitive, and behavioral systems undergo rapid maturation and reorganization. Between the ages of 4 and 6 years, children demonstrate substantial advances in gross motor coordination, executive control, attentional regulation, and sensorimotor integration (1, 2). These domains are routinely monitored within pediatric developmental surveillance frameworks because early weaknesses in motor competence and self-regulatory functioning have been associated with elevated risk for later academic underachievement, behavioral dysregulation, developmental coordination disorder, and attention-deficit/hyperactivity disorder (3–5). Accordingly, identifying modifiable environmental exposures that may strengthen early developmental trajectories remains an important priority in preventive pediatric research.

Gross motor competence is increasingly recognized not only as an indicator of physical capability but also as a potential marker of broader neurodevelopmental status. Children with lower motor proficiency are more likely to exhibit executive function difficulties, reduced self-regulation, and challenges in social participation (5, 6). Structured physical-activity interventions delivered in preschool settings consistently demonstrate improvements in locomotor and object-control skills, with meta-analytic evidence reporting moderate to large behavioral effects (7). Although behavioral efficacy is well established, considerably less is known about whether improvements in motor competence are accompanied by measurable changes in functional neural systems supporting executive and attentional processes. In particular, few randomized preschool trials have examined behavioral and neurophysiological outcomes within the same experimental framework.

From a developmental neuroscience perspective, motor and cognitive systems are functionally interconnected during early childhood. The cerebellum and prefrontal cortex exhibit parallel maturation patterns, and functional connectivity between motor and association networks strengthens markedly across the preschool years (1). Beyond its classical motor role, the cerebellum contributes to executive control, attentional modulation, and behavioral regulation (8). Theoretical accounts grounded in embodied cognition and dynamic systems theory further suggest that repeated, structured motor experiences may influence neural network refinement through experience-dependent processes (4, 9). Despite this theoretical foundation, experimental evidence linking structured motor training to concurrent functional neurodevelopmental indicators in typically developing preschool populations remains limited.

Beyond educational implications, motor competence occupies a recognized position within pediatric developmental monitoring frameworks, where delays in gross motor coordination and self-regulatory behaviors often prompt further evaluation for neurodevelopmental disorders (10). Understanding whether structured motor enrichment strengthens motor competence and is associated with favorable neurodevelopmental indicators therefore carries relevance for preventive pediatric practice. If scalable motor-focused programs delivered within routine kindergarten settings demonstrate measurable developmental benefits, they may represent a feasible population-level strategy to support early developmental trajectories prior to the emergence of clinically significant impairments. Furthermore, identifying functional neural correlates associated with motor enrichment may inform future research aimed at refining early risk detection models, complementing existing behavioral surveillance approaches used in pediatric care.

Therefore, the objective of the present trial is to evaluate the effects of a semester-long motor-focused physical education program on gross motor competence and exploratory neurodevelopmental indicators in preschool children. Based on existing behavioral and developmental neuroscience literature, we hypothesize that children receiving the motor-focused intervention will demonstrate greater improvements in gross motor competence than those in the control condition, exhibit changes in pre-specified electroencephalography (EEG) indices associated with inhibitory control and attentional regulation, and show positive associations between gains in motor competence and changes in functional neural indicators. By integrating standardized behavioral assessment with non-invasive measures of functional brain activity within real-world kindergarten settings, this study aims to clarify whether structured motor enrichment is accompanied by concurrent neurodevelopmental correlates during a sensitive developmental window.

## 2 Methods and analysis

### 2.1 Study design

**Figure 1:**
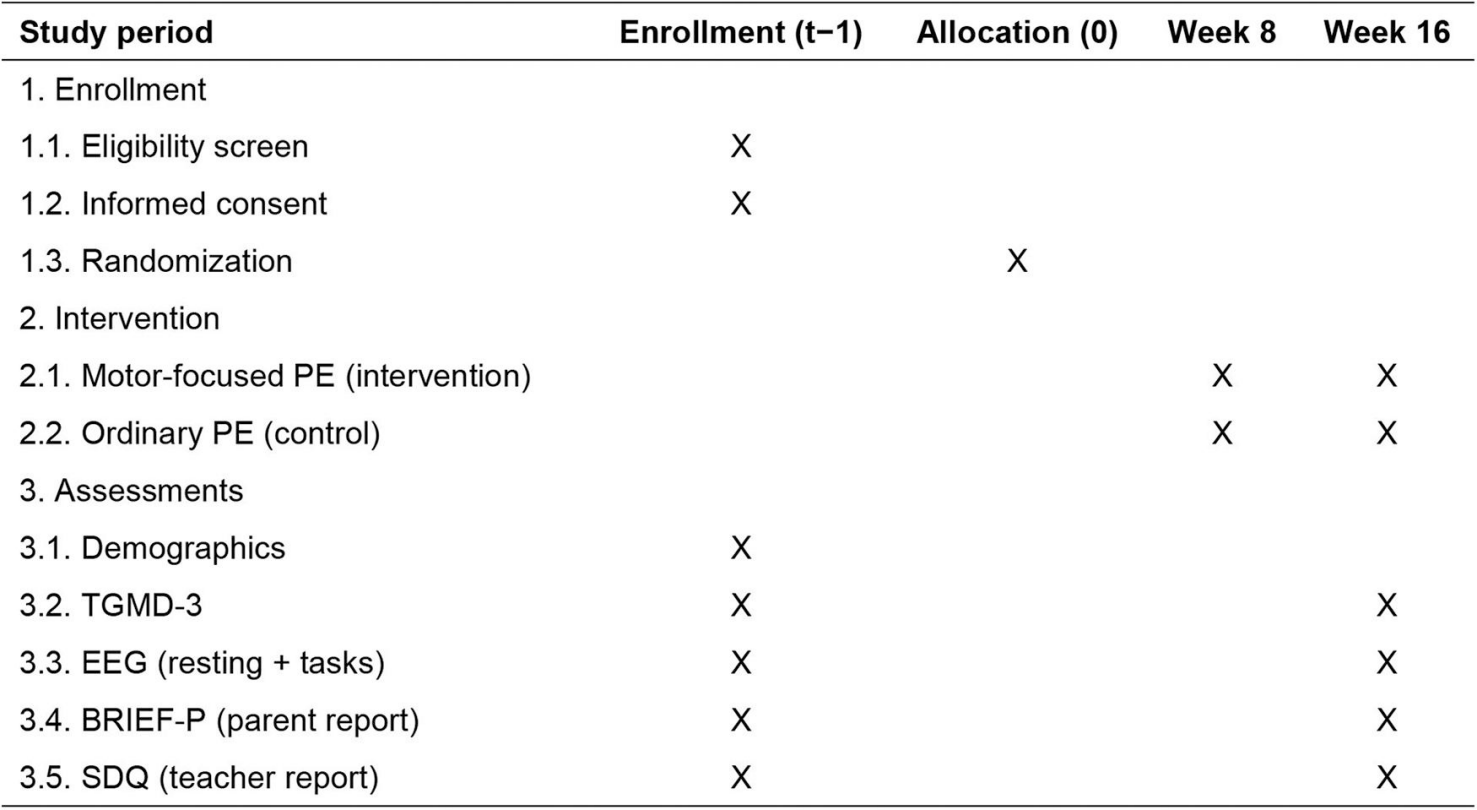
Schedule of enrolment, interventions, and assessments. TGMD-3, test of gross motor development, third edition; EEG, electroencephalography; BRIEF-P, Behavior Rating Inventory of Executive Function-Preschool Version; SDQ, Strengths and Difficulties Questionnaire.

This study is a prospective, two-arm, parallel-group, cluster-randomized controlled trial designed to evaluate the effects of a semester-long (approximately 16 weeks) motor-focused physical education program on gross motor competence and neurodevelopment in preschool children. The study will be conducted in kindergartens from Changsha, Hunan Province, China. The trial follows the recommendations of the SPIRIT 2025 statement for standardized protocol reporting (11). As shown in Figure 1, all participants will undergo eligibility screening, informed consent, and baseline assessments prior to randomization (week 0). The intervention phase spans 16 weeks, during which classes receive either structured motor-focused physical education or ordinary physical education. Outcome assessments are scheduled at baseline (week 0) and immediately post-intervention (week 16). This schedule ensures standardized evaluation and facilitates examination of both motor and neural effects of the intervention.

### 2.2 Participants

Children eligible for this study will be those enrolled in participating kindergartens and aged between 4 and 6 years at the time of recruitment. Participants must be physically healthy and able to engage safely in structured physical education activities. Only children whose parents or legal guardians provide written informed consent will be included.

Children will be excluded if they (1) have diagnosed neurological, developmental, or musculoskeletal disorders that may interfere with motor performance or EEG measurement; (2) are currently enrolled in specialized sports or motor-training programs outside the kindergarten curriculum; or (3) are unable to comply with EEG assessment procedures due to behavioral or sensory intolerance. Children with mild, well-managed conditions (e.g., attention deficit) will not be excluded.

Recruitment will be carried out in collaboration with kindergarten administrators and teachers. Parents will receive written and verbal information about the study objectives, procedures, and potential benefits and risks. Recruitment meetings will be held in participating school to address questions from parents and staff. Consent forms will be distributed to parents, and only children with returned signed consent will be enrolled.

### 2.3 Sample size

The sample size was calculated to detect meaningful differences in gross motor competence between the intervention and control groups, while accounting for the cluster-randomized design. Previous intervention trials and meta-analyses of preschool motor-skill programs have consistently reported large effect sizes, often exceeding 1.0, for improvements in fundamental motor skills (7). Based on this evidence, we adopted an assumption of an effect size of 1.0 for between-group differences.

Using a two-tailed alpha of 0.05 and a statistical power of 80%, an individually randomized design would require approximately 21 children per group. Because this study uses class-level randomization, the required sample size was adjusted for clustering. Assuming an average class size of 25 and an intra-class correlation coefficient of 0.05, the design effect is estimated at 2.2. This adjustment results in an effective sample size of 46 children per group, or 92 children in total. To accommodate an anticipated dropout rate of 15%, we plan to recruit approximately 110 children overall, distributed across four to six kindergarten classes in Changsha city.

### 2.4 Randomization and blinding

Randomization will be performed at the class level. After baseline assessments are completed, participating classes will be randomly assigned in a 1:1 ratio to either the intervention group (motor-focused physical education) or the control group (ordinary physical education). The allocation sequence will be generated by an independent researcher using a computer-based random number generator and will not be disclosed to the assessment team until all baseline testing has concluded.

Due to the nature of the intervention, it is not possible to blind teachers or participants to group allocation. However, outcome assessors will remain blinded throughout the study. Specifically, individuals conducting the motor skill assessments and EEG recordings will not be informed of class assignments. To further reduce bias, video recordings of motor skill performances will be scored by two independent raters who are blinded to intervention status and assessment time point. Data analysts will also be blinded to group labels until the primary analyses are completed.

### 2.5 Intervention

Children assigned to the intervention arm will receive a structured, motor-focused physical education program designed to strengthen fundamental motor skills through progressive, developmentally appropriate movement experiences. The program will be delivered three times per week for approximately 30 to 40 min per session over one academic semester (16 weeks). Each session will follow a standardized structure consisting of a warm-up phase, a structured skill-development phase, and a cool-down period.

Sessions will begin with a 5–7 min warm-up consisting of light locomotor activities, rhythm-based movements, and dynamic stretching designed to prepare children for physical activity. The main skill-development phase (approximately 20–25 min) will emphasize systematic practice of locomotor skills such as running, hopping, sliding, and jumping, as well as object-control skills including throwing, catching, striking, and kicking. Activities will be organized through small-group drills, obstacle courses, and skill stations that progressively increase in complexity across the intervention period. A short game-based integration phase (approximately 5–8 min) will allow children to apply practiced skills within simplified movement games designed to promote coordination, agility, and cooperative interaction. Finally, sessions will conclude with a 3–5 min cool-down, including light stretching and rhythm-based relaxation activities.

The intervention will be delivered by regular kindergarten teachers who will undergo structured preparatory training prior to study initiation. Teachers in the intervention arm will participate in workshop sessions led by the research team, during which they will receive detailed lesson manuals outlining session objectives, activity progressions, instructional cues, and safety procedures. Standardized written materials will be provided to promote consistency across classrooms.

To strengthen intervention fidelity, multiple monitoring procedures will be implemented. Trained research staff will conduct biweekly on-site observations using a standardized fidelity checklist assessing adherence to session structure, accurate delivery of prescribed activities, progression of task difficulty, and child engagement. Each observed session will receive a quantitative adherence score reflecting the proportion of prescribed components implemented as intended. When deviations from protocol are identified, structured feedback will be provided promptly to teachers, and additional guidance or refresher instruction will be arranged if necessary. In addition, a randomly selected subset of sessions will be video-recorded and independently reviewed by members of the research team to verify fidelity ratings and ensure inter-rater consistency. Fidelity data will be summarized and reported in trial outcomes to enhance transparency.

To monitor exercise intensity and ensure that overall physical activity exposure remains comparable between groups, heart rate (HR) will be measured in a randomly selected subsample of children using wearable heart-rate monitors (Huawei Band 10, Huawei Technologies, Shenzhen, China). In each participating class, five children will be randomly selected to wear the device during selected physical education sessions throughout the intervention period. HR will be recorded continuously during the session, providing an objective estimate of exercise intensity and allowing verification that activities reach moderate-to-vigorous physical activity levels typical of preschool physical education. Monitoring will be conducted in both intervention and control classes to ensure comparable overall cardiovascular exertion.

Children assigned to the control arm will continue to receive the standard kindergarten physical education curriculum delivered by their regular teachers. In participating schools, ordinary physical education typically consists of rhythmic exercises, unstructured free play, and simple group games designed to promote enjoyment and social interaction. These sessions do not follow a structured progression targeting specific locomotor or object-control competencies and do not include standardized motor-development lesson plans.

The frequency and duration of control sessions will match those of the intervention group (three sessions per week, 30–40 min per session) to ensure comparable exposure time. Teachers assigned to control classes will not receive motor-focused training workshops, structured lesson manuals, or feedback related to the intervention program. Classes within participating kindergartens are administratively distinct, and teaching staff do not rotate between intervention and control classes during the study period. Intervention materials will be provided exclusively to teachers in the intervention arm, and research staff responsible for fidelity monitoring will not provide instructional guidance to control teachers. These procedures are intended to minimize contamination while preserving the ecological validity of the school-based design.

### 2.6 Outcomes and data collection procedures

The primary outcome of this study will be children's gross motor competence, assessed using the Test of Gross Motor Development, Third Edition (TGMD-3). This standardized instrument provides separate scores for locomotor and object-control skills, as well as a composite gross motor quotient. The TGMD-3 is widely used in early childhood research and has demonstrated strong reliability and validity in preschool populations (12). Assessments will be conducted at baseline and again following the 16-week intervention period in familiar school environments. Children will complete locomotor and object-control subtests under standardized instructions provided by trained assessors. Performances will be video-recorded and subsequently scored by blinded raters.

Secondary outcomes will include neurodevelopmental indicators measured using portable EEG recorded with the BrainLink Lite system (Macrotellect, Shenzhen, China). The device uses dry-electrode technology and records EEG activity from a single frontal electrode positioned at Fp1 according to the international 10–20 system, with reference and ground provided via an ear-clip electrode. Signals are sampled at 512 Hz and transmitted wirelessly to a recording computer via Bluetooth. Similar portable EEG systems have been used in previous research examining cognitive and affective processes in naturalistic settings (13).

Although multi-channel research-grade EEG systems provide higher spatial resolution, their use in preschool populations is often constrained by long preparation times, the need for conductive gels, and reduced tolerance among young children in school environments. Portable single-channel systems therefore offer a practical alternative for field-based developmental research where participant comfort and compliance are critical. The frontal electrode location (Fp1) captures activity from prefrontal regions involved in executive control and attentional regulation, allowing extraction of frontal spectral indices and event-related potentials (ERP) components. Accordingly, the present study adopts a portable frontal EEG configuration to balance methodological rigor with feasibility in a kindergarten setting.

EEG recordings will be obtained during both resting-state conditions and during an age-appropriate cognitive paradigm designed to assess inhibitory control. Resting-state recordings will be conducted while children sit quietly for approximately one minute with minimal movement. Task-related EEG will be recorded during a simplified Go/No-Go paradigm adapted for preschool children, which has been widely used to assess early inhibitory control and attentional regulation during the preschool years.

To enhance interpretability and avoid exploratory overreach, the trial specifies a limited set of pre-defined neural indices. The primary EEG outcome will be frontal theta power measured during resting state. Frontal theta activity has been associated with functional engagement of prefrontal control systems and demonstrates developmental sensitivity across early childhood (14, 15). A secondary task-related neural index will be the N2 component elicited during the Go/No-Go paradigm. The N2 has been widely interpreted as a neural indicator of conflict monitoring and response inhibition and has been reliably elicited in preschool-aged children using simplified Go/No-Go paradigms (16–18). Because inhibitory control represents a foundational element of executive functioning that is routinely considered within pediatric developmental assessment frameworks, these neural measures are selected as developmentally interpretable functional indicators rather than as direct measures of neural plasticity or structural brain change.

Additional EEG measures will include spectral power indices in the alpha and beta frequency bands to provide complementary information regarding cortical activation patterns. Behavioral performance during the Go/No-Go task, including response accuracy and reaction time, will be analyzed alongside EEG indices to contextualize neural findings. Exploratory analyses will examine associations between changes in gross motor competence and changes in EEG-derived neural indicators. These exploratory analyses are intended to generate hypotheses for future research rather than to establish definitive mechanistic pathways.

EEG data preprocessing will follow standardized procedures for portable EEG recordings. Raw signals will first undergo 50 Hz notch filtering to remove electrical line noise, followed by bandpass filtering between 0.5 and 45 Hz. Data will then be segmented into task-related epochs and corrected using baseline activity from the resting-state recording. Segments contaminated by excessive movement, ocular activity, or muscle artifacts will be identified using amplitude thresholds (e.g., ±80–100 μV) and removed. Remaining trials will be visually inspected by trained analysts to ensure data quality before averaging. Spectral power will be calculated using fast Fourier transform methods, and ERP components such as the N2 will be extracted from averaged task-related waveforms. Preprocessing will be conducted by research staff blinded to group allocation.

Additional measures will be included to provide complementary perspectives on children's everyday functioning beyond structured testing. Parents will complete the Behavior Rating Inventory of Executive Function–Preschool Version (BRIEF-P) (19), which assesses domains including inhibition, shifting, emotional control, working memory, and planning/organization. Teachers will complete the Strengths and Difficulties Questionnaire (SDQ) (20), which evaluates behavioral adjustment, prosocial behavior, hyperactivity, and peer relationships. Questionnaires will be administered at baseline and post-intervention via secure electronic survey. These instruments will serve as supplementary outcomes to provide ecologically valid information regarding children's executive and socio-emotional functioning in daily contexts.

### 2.7 Data management and quality assurance

All data will be managed in accordance with institutional and international standards for research involving human participants. Each child will be assigned a unique identification code, and all data will be labeled with this code rather than personal identifiers to protect confidentiality. Video files of TGMD-3 performances and EEG recordings will be stored on encrypted, password-protected hard drives accessible only to the research team. Questionnaire data will be entered into a secure database.

TGMD-3 scoring will be completed by two independent raters who are blinded to both group allocation and time point. Inter-rater reliability will be assessed using intraclass correlation coefficients, and any discrepancies will be resolved by consensus discussion. EEG data will be preprocessed using standardized pipelines to minimize noise and artifact, including filtering, segmentation, and removal of movement-related or ocular artifacts. Preprocessing will be performed by trained staff who are blinded to group allocation.

A designated data manager will oversee double-entry checks for questionnaire and TGMD-3 scoring data to ensure accuracy. Any irregularities will be flagged and corrected in consultation with the principal investigator. Interim analyses will not be conducted, but descriptive monitoring of recruitment, retention, and fidelity will be used to ensure study progress.

### 2.8 Statistical analysis

All analyses will follow the intention-to-treat principle, with all children analyzed according to their randomized class regardless of adherence or attendance. Descriptive statistics will be reported for baseline demographic and developmental characteristics, including age, sex, height, weight, and baseline TGMD-3 scores, to assess comparability between groups. Continuous variables will be summarized using means and standard deviations, while categorical variables will be summarized using frequencies and percentages.

For the primary outcome of gross motor competence, linear mixed-effects models will be used to examine group (intervention vs. control) by time (baseline vs. post-intervention) interactions, accounting for clustering at the class level. Models will include random intercepts for class and child, and fixed effects for group, time, and the group-by-time interaction. Relevant covariates, including age and sex, will be included where appropriate. Because the number of clusters (classes) in the present study is relatively small, degrees of freedom for fixed effects will be estimated using the Satterthwaite approximation within the linear mixed-effects modeling framework. This approach reduces potential underestimation of standard errors and helps control Type-I error rates in studies with limited cluster numbers.

For EEG outcomes, analyses will follow the pre-specified hierarchy of neural endpoints. The primary EEG analysis will focus on frontal theta power during resting state. A secondary task-related neural index will be the N2 amplitude elicited during the Go/No-Go task. Separate linear mixed-effects models will be constructed for each neural endpoint to evaluate group-by-time interactions, using the same clustering structure as described for the primary motor outcome (random intercepts for class and child).

Additional EEG measures will include spectral power indices in the alpha and beta frequency bands, which will be analyzed using similar mixed-effects models. To account for multiple comparisons across these secondary neural measures, false discovery rate adjustments will be applied. These analyses will be interpreted as supportive and contextual rather than confirmatory.

Exploratory analyses will examine associations between changes in TGMD-3 scores and changes in EEG-derived neural indicators, as well as feasibility analyses of EEG recordings obtained during selected motor tasks. Correlational analyses will use Pearson or Spearman coefficients as appropriate, with regression models applied to adjust for relevant covariates. Results from exploratory analyses will be interpreted as hypothesis-generating.

Behavioral performance measures from cognitive tasks, including accuracy and reaction time, will be analyzed using mixed-effects models parallel to those used for neural indices. Questionnaire outcomes (BRIEF-P and SDQ) will also be examined using mixed-effects models to compare baseline-to-post changes across groups, adjusting for clustering.

Missing data for behavioral and questionnaire outcomes will be handled using maximum likelihood estimation within the linear mixed-effects modeling framework, which provides unbiased parameter estimates under the assumption of missing at random (MAR). This will serve as the primary approach to incomplete outcome data. The proportion and pattern of missingness will be examined descriptively. If missingness exceeds anticipated levels or suggests deviation from the MAR assumption, sensitivity analyses will be conducted to evaluate the robustness of findings.

All statistical tests will use a two-sided alpha level of 0.05. Analyses will be performed using SPSS 25.0 (IBM, Armonk, NY, USA).

## 3 Discussion

This trial is designed to evaluate whether a structured motor-focused physical education program improves gross motor competence and is associated with concurrent changes in functional neurodevelopmental indicators in preschool children. While previous research has consistently demonstrated improvements in gross motor skills following structured motor interventions, less is known about how such training relates to functional brain activity during early childhood. By integrating portable EEG measures within a cluster-randomized design, the present study seeks to examine behavioral and neurodevelopmental outcomes within the same experimental framework.

A major strength of this trial lies in its ecological validity. The intervention will be delivered in real-world kindergarten classrooms by regular teachers using standardized lesson materials, enhancing the practical relevance of the findings. The use of cluster randomization, blinded motor assessments, structured fidelity monitoring, and predefined neural endpoints strengthens internal validity while maintaining feasibility in educational settings. The inclusion of EEG measures allows the examination of functional neural indicators associated with executive and attentional processes; however, these measures are intended to complement behavioral findings rather than to establish definitive mechanistic explanations.

The trial incorporates multiple levels of outcome assessment, including standardized motor testing (TGMD-3), neurophysiological indices, and ecologically valid parent- and teacher-reported measures. This multi-method approach permits examination of whether behavioral improvements in motor competence are accompanied by changes in functional indicators of self-regulatory processes. Nevertheless, neural findings will require cautious interpretation. EEG markers such as frontal theta activity and N2 amplitude are developmentally interpretable within early childhood research, but they do not provide direct evidence of structural brain change or long-term neurodevelopmental modification.

Several limitations warrant consideration. First, although portable EEG enables assessment in preschool environments, recordings are inherently vulnerable to movement artifacts and compliance challenges. Despite standardized preprocessing and artifact rejection procedures, some data loss is anticipated. Second, while the study is powered to detect large effects on the primary behavioral outcome, it is not powered for definitive inference regarding neurophysiological endpoints. Accordingly, EEG analyses should be interpreted as exploratory and hypothesis-generating. Third, the intervention duration is limited to one academic semester within a single regional context, and longer-term developmental trajectories cannot be inferred from the present protocol.

Despite these limitations, this study has the potential to make several important contributions. It will provide one of the first randomized controlled trials to integrate EEG into the evaluation of early motor-focused physical education, thereby linking behavioral and neural outcomes in a developmental framework. It will also generate practical insights for educators and policymakers by identifying not only whether motor-focused programs are beneficial, but also how they exert their influence on the developing brain. Ultimately, this knowledge may inform the design of optimized physical education curricula that promote both motor competence and cognitive readiness in young children, with long-term implications for academic achievement, physical activity participation, and health across the lifespan.

Within these boundaries, the study aims to contribute incremental evidence to the growing literature linking motor development and neurocognitive functioning in early childhood. The findings are intended to clarify whether structured motor-focused programs are associated with concurrent functional neural indicators during a sensitive developmental period. If such associations are observed, they may inform future research examining scalable approaches to support early developmental trajectories within educational settings.

## 4 Ethics and dissemination

This study protocol has been reviewed and approved by the Ethics Committee of Hunan Normal University (approval number: 2025-532). All procedures will be conducted in accordance with the ethical principles outlined in the Declaration of Helsinki. The trial has been prospectively registered at ClinicalTrials.gov (identifier: NCT07307742, date of registration: December 26, 2025).

Written informed consent will be obtained from the parents or legal guardians of all participating children, and assent will be sought from children in an age-appropriate manner. Participation is voluntary, and families may withdraw at any time without penalty. Data confidentiality will be strictly maintained by assigning anonymized codes to all participants and storing information on secure, password-protected servers. Only the research team will have access to the data.

The risks associated with participation are minimal. Structured physical education activities are age-appropriate, consistent with typical preschool play, and will be supervised by trained teachers. EEG recording is non-invasive and will be conducted in a child-friendly manner to minimize discomfort or fatigue. The expected benefits include improvements in children's motor competence and developmental experiences, as well as contributions to advancing scientific knowledge.

Recruitment is expected to begin in March 2026 and continue until June 2026. Baseline assessments and randomization will take place immediately after enrollment, with the 16-week intervention phase running from March to July 2026. Post-intervention assessments are scheduled for July–August 2026. Data analysis will begin in September 2026, and the first results are anticipated for dissemination in late 2026 or early 2027.

Findings from this trial will be disseminated through peer-reviewed publications, presentations at national and international conferences, and lay summaries prepared for participating schools and families. Results will also be shared with local education authorities and policy stakeholders to inform evidence-based recommendations for early childhood physical education.
